# Association of the *STAT4* Gene rs7574865 Polymorphism with IFN-γ Levels in Patients with Systemic Lupus Erythematosus

**DOI:** 10.3390/genes14030537

**Published:** 2023-02-21

**Authors:** Yussef Esparza Guerrero, Maria Luisa Vazquez Villegas, Cesar Arturo Nava Valdivia, Juan Manuel Ponce Guarneros, Edsaul Emilio Perez Guerrero, Eli Efrain Gomez Ramirez, Melissa Ramirez Villafaña, Betsabe Contreras Haro, Alejandra Martinez Hernandez, Ernesto German Cardona Muñoz, Ismael Nuño Arana, Sergio Gabriel Gallardo Moya, Alfredo Celis, Laura Gonzalez Lopez, Jorge Ivan Gamez Nava, Ana Miriam Saldaña Cruz

**Affiliations:** 1 Programa de Doctorado en Farmacología, Departamento de Fisiología, Centro Universitario de Ciencias de la Salud, Universidad de Guadalajara, Guadalajara 44340, Mexico; 2 Departamento de Epidemiología, Unidad de Medicina Familiar No. 4, Instituto Mexicano del Seguro Social (IMSS), Guadalajara 44340, Mexico; 3 Instituto Regional de Investigación en Salud Pública, Programa de Doctorado y Coordinación del Programa de Doctorado en Salud Pública, Departamento de Salud Pública, Centro Universitario de Ciencias de la Salud, Universidad de Guadalajara, Guadalajara 44340, Mexico; 4 Departamento de Microbiología y Patología, Centro Universitario de Ciencias de la Salud, Universidad de Guadalajara, Guadalajara 44340, Mexico; 5 Departamento de Fisiología, Centro Universitario de Ciencias de la Salud, Universidad de Guadalajara, Guadalajara 44340, Mexico; 6 Instituto de Investigación en Ciencias Biomédicas, Centro Universitario de Ciencias de la Salud, Universidad de Guadalajara, Guadalajara 44340, Mexico; 7 Instituto de Terapéutica Experimental y Clínica, Departamento de Fisiología, Centro Universitario de Ciencias de la Salud, Universidad de Guadalajara, Guadalajara 44340, Mexico; 8 Unidad de Investigación Biomédica 02, UMAE, Hospital de Especialidades, Centro Médico Nacional de Occidente, IMSS, Guadalajara 44349, Mexico; 9 Departamento de Ciencias Biomédicas, Centro Universitario de Tonalá, Universidad de Guadalajara, Guadalajara 45400, Mexico

**Keywords:** systemic lupus erythematosus, STAT4, IFN-gamma, polymorphism

## Abstract

STAT4 plays an important role in disease activity in SLE patients. STAT4 particles have the capacity to activate the transcription of genes associated with the production of TH1 and Th17 lymphocytes, with a greater predominance on the production of IFN-γ and IL-17A. The presence of variants in *STAT4* genes has a major impact on the generation of autoimmunity. However, there are few studies evaluating the impact of these variants on the production of proinflammatory cytokines such as IFN-γ and IL-17A. Methods—A case–control study was carried out with 206 Mexican mestizo patients residing in Western Mexico with a diagnosis of SLE and a group of 80 patients without autoimmune diseases was captured to determine the cut-off point for high IFN-γ levels. In this study, SLE patients with high IFN-γ levels were considered as cases (cut-off > 15.6 pg/mL), and SLE patients with normal IFN-γ levels were considered as controls (cut-off ≤ 15.6 pg/mL). Disease activity was identified from the systemic lupus erythematosus disease activity index (SLEDAI). For the determination of levels of cytokines IFN-γ, IL-12, and IL17A, commercial ELISA kits were used. Genotyping of STAT4 rs7574865 (G > T) was performed by quantitative polymerase chain reaction (qPCR) using TaqMan probes. Results—The patients with SLE had a median age of 45 years with a range of disease duration from 4 years to 18 years; 45.6% were identified as having disease activity. In this sample, we identified a high IFN-γ prevalence of 35.4%. The levels of IFN-γ were higher in the patients with genotype TT than GG. We found that TT genotype conferred a higher risk of high IFN-γ when compared to the GG and GT genotypes. Conclusions—In this study, we identified that the polymorphic genotype TT of the *STAT4* gene rs7574865 polymorphism is associated with increased levels of IFN-γ. However, its strength of association was weak, so complementary studies are needed to evaluate its impact on SLE patients.

## 1. Introduction

Systemic lupus erythematosus (SLE) is a chronic, autoimmune disease, characterized by the presence of inflammatory activity in multiple organs and systems [1,2]. This disease is characterized by the presence of immunocomplexes in different tissues, generating complement activation and an extensive inflammatory response [3].

SLE was found to have a different interferon (IFN) gene signature [4]. Interferon is a cytokine produced in response to viral infection and has other actions, such as regulating immunity, antitumor, and antiviral activities. IFNs are divided into three types [5]: INF type I, which include IFN-α subtypes and IFN-β, -ϵ, -κ, and -ω; type II, which includes IFN-γ; and type III, which includes IFN-λ [6]. The interferon-γ (IFN-γ) cytokine is produced by stimulation of IL-12, which mediates TH1 lymphocyte differentiation [7]. One of its main effects is B lymphocyte differentiation into autoantibody-producing cells and the activation of macrophages [8,9,10]. IL-17A is a proinflammatory cytokine produced by activated T lymphocytes; it has an important proinflammatory effect by activating the chemotaxis of monocytes and neutrophils, increasing the production of other proinflammatory cytokines and adhesion molecules, as well as generating greater production of autoreactive antibodies [11,12].

The convergent pathway in the production of a large group of cytokines is dependent on JAK/STAT signaling, which is a group of intracellular messengers that increment cellular differentiation. The ligand-dependent activation of JAK generates a phosphorylation cascade that activates STAT, which translocates to the nucleus and activates the transcription of genes related to cell differentiation and cytokine production [13]. In the case of IFN-γ and IL-17A, in particular, their production depends on the activation of Signal Transducer Activator of Transcription 4 (STAT4) proteins [13,14].

STAT4 is part of the Janus Kinase (JAK)–STAT pathway. There are seven members of the STAT family (STA1, STAT2, STAT3, STAT4, STAT5, STAT6, and STAT7) that are involved in innate and adaptative immune responses [15]. STAT4 comprises an N-terminal domain that plays an important role in phosphorylation and nuclear translocation, as well as a four-stranded helical coil that is implicated in protein–protein interactions and nuclear import and export [16]. STAT4 can activate the transcription of genes associated with the production of TH1 and Th17 lymphocytes, with a greater predominance of the production of IFN-γ and IL-17A [17].

The *STAT4* gene is located on human chromosome 2q32.2-q32.3, consists of 27 exons, and encodes a transcription factor that is expressed in dendritic cells, macrophages, and lymphocytes [18]. STAT4 is a critical mediator of the immune response; any changes in the activity of the protein or in the expression leads to alterations in the immune system response and function. For instance, the rs7574865 polymorphism of the *STAT4* gene is a G > T change in the third intron, which has been associated with its TT polymorphic genotype with the increased presence of autoimmunity. In patients with SLE, the polymorphic variant has been identified as being associated with increased susceptibility to presenting with the disease, as well as an increased risk of presenting positive autoantibodies [19,20].

Many studies have investigated the relevance of the STAT4 polymorphism as a potential factor associated with SLE susceptibility, but none of them have evaluated the impact of the polymorphic variant’s presence with the serum levels of IFN-γ [21]. Only one study tried to explore the effect of the STAT4 polymorphism in the cellular and serum immune response, without evidence of the association with IFN-γ levels. Hagberg et al. observed, in Swedish patients with SLE, that patients with the TT polymorphic genotype had higher levels of IFN-γ in plasma with non-statistical differences [15].

Therefore, the aim of this study is to evaluate the association between different genotypes of the rs7574865 polymorphism of the *STAT4* gene with the serum levels of IFN-γ in patients with SLE.

## 2. Methodology

Study design: A case-control study was carried out on patients with a diagnosis of SLE. In order to be included in the study, patients fulfilled the inclusion criteria of an SLE diagnosis according to the 1982 American College of Rheumatology criteria and were Mexican mestizo patients according to the Mexican National Institute of Anthropology and History (INAH), residing in Western Mexico [22]. Patients who underwent blood transfusions in the last 3 months, had overlap syndromes, had a diagnosis of acute or chronic disease, and who were pregnant were excluded. 

A group of 80 patients without autoimmune diseases was captured to determine the cut-off point for IFN-γ levels, which is considered as the 75th percentile or higher. In this study, a high cut-off point for IFN-γ was identified as 15.6 pg/mL. In this study, SLE patients with high IFN-γ levels were considered as cases (cut-off > 15.6 pg/mL), and SLE patients with normal IFN-γ levels were considered as controls (cut-off ≤ 15.6 pg/mL).

SLE patients and the control group were assessed by trained researchers, using a structured interview recording the epidemiological and clinical characteristics of the disease, such as disease duration, history of smoking, and alcohol consumption. After 8-h of fasting, a venous sample was obtained from the patients (SLE and without autoimmune disease group).

Clinical evaluations: Disease activity was evaluated by trained researchers using the systemic lupus erythematosus disease activity index (SLEDAI). This index is a validated instrument to evaluate disease activity. It consists of the evaluation of 9 organs or systems considering the last 10 days before its evaluation. This index is scored through 24 items, which generate a score between 0 and 105. SLE activity was defined as SLEDAI score ≥4 points [23].

Cytokine determinations: For the determination of cytokines (IFN-γ, IL-12, and IL-17A), blood was drawn by venipuncture and then centrifuged at 1300 rpm for 15 min at 4 °C. IFN-γ levels were quantified with a commercial sandwich ELISA kit (MyBioSource, Inc., San Diego, CA, USA). The sensitivity of this assay was 9.38 pg/mL, range detection 15.6 to 1000 pg/mL, and intra-assay precision <8%. IL-12 was determined with an ELISA kit (Biovendor, Czech Republic) with a sensitivity of <10 pg/mL, range detection 15.6 to 1000 pg/mL, and intra-assay precision <10%, and IL17A with an ELISA kit (Invitrogen, Camarillo, CA, USA) with a sensitivity of 7.5 pg/mL, range detection 4 to 500 pg/mL intra-assay, and precision <9.1%.

Genotyping: Peripheral whole blood samples were obtained in vacutainer tubes that contained ethylenediaminetetraacetic acid as an anticoagulant (EDTA) from the SLE patients. DNA was extracted from the lymphocytes. Genomic DNA extraction was performed using the modified Miller’s technique [24].

After the genomic DNA was obtained, it was quantified using a Nanodrop Genomic; the DNA was diluted in a Tris-EDTA buffer to 20 ng/μL and placed in 200 μL polyropylene cryotubes (Eppendorf™). Genotyping of STAT4 rs7574865 was performed by quantitative polymerase chain reaction (qPCR) using TaqMan probes. TaqMan Assay IDs C__29882391_10 was performed according to the manufacturer’s instructions (Applied Biosystems, Waltham, MA, USA); the StepOne™ Real-Time polymerase chain reaction (qPCR) system was employed for this purpose (Applied Biosystems). All results were independently scored by two investigators blinded to patient information. In case of ambiguous results, the sample was analyzed a second time.

Other laboratory determinations: The laboratory assessment included the complement fractions C3 and C4 by dry chemistry (Vitros 3500/4600 analyzer Ortho Diagnostics). We considered low C3 levels under 90 mg/dL, and C4 levels under 10 mg/dL. Antibodies against double-stranded DNA (anti-dsDNA) were measured using a commercial ELISA kit (MyBiosource, Inc., San Diego, CA, USA; assay sensitivity of 1.0 IU/mL). Positivity of anti-dsDNA was defined as a titer ≥ 14 RU/mL

## 3. Statistical Analysis

Quantitative variables were expressed as medians (IQR; interquartile range) and quantitative variables as frequencies (%). A chi-square test was used to compare qualitative variables, and the Mann–Whitney U test was used for quantitative variables. For the comparison of serum cytokine levels between genotypes, a Kruskal–Wallis test was used. Odds ratios (ORs) with 95% confidence intervals (95% CIs) were used for risk estimation. We used a multivariable analysis to determine which variables are risk factors for high IFN-γ, and two multivariable logistic regression models were developed. The first model was used for the estimation crude ORs using the enter method. In the second model, we estimated adjusted ORs using the forward stepwise method. The covariates for these models were variables that were significant in the univariable analyses or variables that had biological plausibility for increasing the potential risk of developing the dependent variables. Adjusted ORs and their 95% CIs. Statistical significance was considered as a *p*-value ≤ 0.05. The data was analyzed with SPSS v29 software. The graphs were made using GraphPad Prism 9.

## 4. Ethics and Consent

This study was approved by the local research and ethics committee of the Mexican Institute of Social Security (IMSS) with the registration number R-2019-1305-075. The study adhered to the guidelines of the Declaration of Helsinki. All subjects agreed to participate on a voluntary basis and signed an informed consent form.

## 5. Results

We selected 206 patients with systemic lupus erythematosus with a median age of 45 years and with a IQR of disease duration from 4 years to 18 years. In addition, of the evaluated samples of patients, 45.6% were identified as having disease activity at the time of the study, identified as a SLEDAI score of ≥4. The main type of activity present in patients was identified as mucocutaneous activity in 21.4% of patients, followed by renal activity in 20.4% and central nervous system activity in 10.7% (Table 1).

Within the population studied, 87.4% of patients presented with glucocorticoid use. In addition, 76.2% were prescribed immunosuppressants at the time of the study, with the most commonly used immunosuppressant being azathioprine.

A high interferon score was determined, in which 35.4% of the patients were found to have it. Table 1 shows the cytokine levels. In relation to the *STAT4* gene rs7574865 polymorphism genotypes, it was found that 31.6% presented with the wild homozygous genotype (GG), 48.5% presented with a heterozygous genotype, and only 19.9% of patients presented with the polymorphic homozygous genotype.

In the comparison between groups of patients with high and low interferon, it was identified that there were differences in the time of evolution of the disease. No differences were identified in the rest of the characteristics. We can observe that the IL-17A levels are lower in the group of patients with high IFN-γ (Table 2). In the comparation of the pharmacological treatments, no differences were identified between patients with high IFN-γ and normal IFN-γ. 

**Figure 1:**
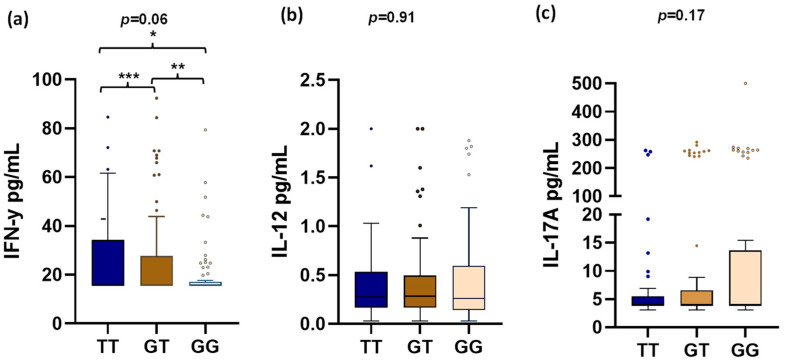
Comparison of serum IFN-γ, IL-12, and IL-17A levels between genotypes of the rs7574865 polymorphism. Cytokine levels are reported in pg/mL, expressed as median and IQR (interquartile range). (**a**) Shows the comparation of IFN-γ serum levels into different genotypes of rs7574865 polymorphism *STAT4* gene. (**b**) Shows the comparation of IL-12 serum levels into different genotypes of rs7574865 polymorphism *STAT4* gene. (**c**) Shows the comparation of IL-17 A serum levels into different genotypes of rs7574865 polymorphism *STAT4* gene. GG: wild homozygote genotype; GT: heterozygote genotype; TT: polymorphic homozygote. The Kruskall–Wallis test was used to compare the three genotypes. For the comparison between two groups, the Mann–Whitney U test was used. IFN-γ: interferon γ, IL-12: interleukin 12, IL-17A: interleukin 17A. * *p* = 0.018, ** *p* = 0.13, *** *p* = 0.3.

In the comparison between groups, a higher level of IFN-γ was observed in patients with the TT genotype compared to the GG, with a statistically significant difference. When comparing GT vs. TT genotypes, there was a non-significant tendency to present higher levels in the TT genotype (Figure 1).

In the risk analysis, it was observed that the TT genotype conferred a higher risk of presenting high IFN-γ levels when compared against the GG and GT genotype (Table 3). A multivariate analysis was performed, and it was identified that the TT genotype confers a higher risk of presenting high IFN-γ (Table 4).

## 6. Discussion

In this study, we found important information related to the association of the *STAT4* gene rs7574865 polymorphism with IFN-γ levels in Mexican patients with SLE. We observed that patients with the TT genotype presented higher levels of IFN-γ compared to the rest of the genotypes. We found no association between *STAT4* gene polymorphism and IL-12 or IL-17A levels. 

In this study of patients with SLE, we identified that the prevalence of the polymorphism was 19.9% TT (polymorphic genotype), 48.5% GT (heterozygous genotype), and 31.6% GG (wild-type genotype). Our findings are supported by Beltran Ramirez, who found that among the Mexican population with SLE, 26.6% of patients had the TT genotype and 53.1% had the GT genotype, with the remaining patients with the GG genotype comprising 20.3% [25].

We observed higher levels of IFN-γ in patients with the TT polymorphic genotype in the *STAT4* gene. This is the first study that identified this association in Mexican patients with SLE. The only other study that has investigated a possible association in serum levels between the different genotypes of the *STAT4* rs7574865 polymorphism was that performed by Hagberg et al. in 52 Swedish patients with SLE, in which it was observed that patients with the TT polymorphism genotype had higher levels of IFN-γ in plasma without a statistical difference. The Hagberg study was unable to identify an association between the rs7574865 polymorphism of the *STAT4* gene and serum IFN-γ levels. However, in the Hagberg study, only 52 patients were evaluated, of which only nine had the TT polymorphic variant [26].

In this study, we observed that the recessive model (TT vs. GG and GT) rs7574865 polymorphism of the *STAT4* gene confers a risk of high levels of IFN-γ (OR: 2.01, 95% CI: 1.01–4.03, *p* = 0.046). This result has not been published in any other study.

STAT4 is an important component in the differentiation of TH1 and TH17 lymphocytes and, consequently, the production of IFN-γ and IL-17A in patients with SLE. Studies have reported increased STAT4 mRNA and protein expression in patients with an autoimmune disorder, including SLE [17].

The great importance of the STAT4 pathway in the generation of proinflammatory cytokines in patients with SLE proposes a therapeutic basis for the use of Janus Kinase inhibitors in the management of patients with this disease. However, despite this, the results obtained in several clinical trials have not shown sufficient evidence to justify their use [27]. An important theory that could be associated with these results is the presence of genetic variants in the JAK/STAT pathway, which may modify the production of proinflammatory cytokines and, therefore, the therapeutic response.

The rs7574865 polymorphism of the *STAT4* gene has been associated with the presence of increased expression; however, the precise mechanism remains unclear. This polymorphism is located at the third intron of the *STAT4* gene instead of in the promoter region. It is known that introns do not code for the protein, and the recently intensively debated ‘biological function’ should be extended to expression regulation by cis-acting elements located at the outside regions of protein-coding parts in genes [28]. 

The Hagberg study shows evidence of how an intronic SNP in the major STAT4 SLE risk locus affects the function of immune cells for patients with SLE in a cell-type-specific and context-dependent manner, as in the case of IFN-γ [26]. Serum IFN-γ levels are influenced by multiple factors, such as environmental and cellular microenvironment-related factors. Among them, we can recognize ethnicity and lymphocyte count as factors associated with higher levels of IFN-γ, while smoking is associated with lower levels of this cytokine. The identification of factors affecting the production of these proinflammatory cytokines is of vital importance [29]. A strength of this study is that it was carried out in a population of the same ethnic group, defined as Mexican mestizos, according to the definition of the National Institute of Anthropology and History (INAH) [22]. Similarly, the frequency of smoking was studied and showed no differences between the two groups. Other factors that could influence IFN-γ values were controlled through exclusion criteria. 

Another remarkable result in this study is that our patients were stratified to those with “high” levels according to IFN-γ measurements, by estimating the third quartile of IFN-γ values in the patient control group. Our study identified that the group of patients with high interferon levels had lower levels of IL-17A. Several studies have postulated the complex interaction between IFN-γ and IL-17A. They have proposed that interferon has a regulatory effect on this cytokine, generating lower levels of IL-17A; that hypothesis is supported by our results [30,31].

The role of IFN-γ levels in SLE has been studied by others; Qian-Ling et. al. proposed that IFN-γ may serve as auxiliary indexes for SLE diagnosis [32]. Experimental studies have shown that IFN-γ can accelerate SLE, while the anti-IFN-γ antibody and soluble recombinant IFN-γR (sIFNR) can delay the onset of the disease [33]. Thus, overexpression of IFN-γ causes further inflammation and tissue injury and contributes to the immunopathogenesis of SLE.

One of the main weaknesses of case and controls designs is that they cannot demonstrate causality. This criticism also applies to this study; therefore, although these results proposed that the TT genotype polymorphism is associated with serum levels of IFN-γ, it cannot demonstrate causality. 

On the other hand, one of the greatest strengths of this study is that it evaluates the results of cytokine serum levels among the different genotypes of the rs7574865 polymorphism and presents the phenotypic characteristics of patients with SLE. This is the first study to identify this association between serum IFN-γ levels and genotypes of the *STAT4* gene rs7574865 polymorphism. A possible future perspective is to complement these results with in vitro models, where the creation of a cell line with the different genotypes will allow us to identify the possible mechanism to explain the increase in IFN-γ production. Another perspective that future studies can evaluate is the difference in therapeutic response to treatment with Janus Kinase inhibitors in patients with the polymorphic genotype compared to patients with other genotypes of the *STAT4* gene rs7574865 polymorphism.

## 7. Conclusions

In conclusion, this study identified that the rs7574865 polymorphism of the *STAT4* gene is associated with increased levels of IFN-γ. However, its strength of association is weak, so complementary studies are needed to evaluate its impact on SLE patients.

## Figures and Tables

**Table 1:** Characteristics of systemic lupus erythematosus.

Variable	SLE
	(*n* = 206)
Age, years *median (IQR)*	45 (35–54)
Female, *n (%)*	192 (93.2)
Active Smoker, *n (%)*	18 (8.7)
Alcohol Consumption, *n (%)*	27 (13.1)
Disease duration, year *median (IQR)*	10 (4–18)
Organic Affection	
Mucocutaneos, *n (%)*	44 (21.4)
Renal, *n (%)*	42 (20.4)
Central Nervous System, *n (%)*	22 (10.7)
Musculoskeletal, n (%)	15 (7.3)
Hematologic, *n (%)*	5 (2.4)
SLEDAI Score, *median (IQR)*	2 (0–6)
SLEDAI ≥ 4 *n (%)*	94 (45.6)
Low C3, *n (%)*	28 (13.6)
Low C4, *n (%)*	57 (27.7)
Anti-DNAds positive, *n (%)*	46 (22.3)
**Serological Characteristics**	
IFN-γ, *pg/mL median (IQR)*	16 (16–24)
High IFN-γ, ≥15.6 *pg/mL*	73 (35.4)
IL-12, *pg/mL median (IQR)*	0.27 (0.16–0.51)
High IL-12, >0.52 pg/mL *n (%)*	175 (85)
IL-17A, *pg/mL median (IQR)*	4 (4–7)
High IL-17A >7.10 pg/mL, *n (%)*	45 (21.8)
**Genetic Characteristics (Genotyping rs7574865 STAT4)**	
GG Genotype, *n (%)*	65 (31.6)
GT Genotype, *n (%)*	100 (48.5)
TT Genotype, *n (%)*	41 (19.9)
Allelic frequency 2*n* = 412	
G Allele, *n (%)*	230 (55.8)
T Allele, *n (%)*	182 (44.2)

Quantitative variables are expressed in median and IQR (interquartile range), and qualitative in frequency and percentage. SLEDAI: systemic lupus erythematosus disease activity, ANTI-DNAds: anti-DNA double stranded. IL: interleukin. IFN-γ: interferon γ. GG: wild homozygote genotype; GT: heterozygote genotype; TT: polymorphic homozygote.

**Table 2:** Comparison of characteristics of systemic lupus erythematosus in patients with or without high IFN-γ.

Variable	High IFN-γ	Normal IFN-γ	*p*
	(*n* = 73)	(*n* = 133)	
Age, years *median (IQR)*	45 (37–52)	44 (35–54)	0.52
Female, *n (%)*	66 (90.4)	126 (94.7)	0.23
Active Smoker, *n (%)*	6 (8.2)	12 (9.0)	0.84
Alcohol Consumption, *n (%)*	9 (12.3)	18 (13.5)	0.80
Disease duration, year *median (IQR)*	13 (5–20)	8 (4–16)	0.036
SLEDAI Score, *median (IQR)*	2 (0–4)	2 (0–6)	0.33
SLEDAI ≥ 4 *n (%)*	31 (42.5)	63 (48.1)	0.44
Low C3, *n (%)*	12 (16.4)	16 (23.2)	0.73
Low C4, *n (%)*	28 (38.6)	29 (46.0)	0.46
Anti-DNAds positive, *n (%)*	19 (26.0)	27 (40.9)	0.42
**Serological Characteristics**			
IL-12, *pg/mL median (IQR)*	0.24 (0.13–0.43)	0.29 (0.17–0.56)	0.12
High IL-12, >0.52 pg/mL *n (%)*	61 (85.9)	114 (93.4)	0.08
IL-17A, *pg/mL median (IQR)*	3.9 (3–5)	3.91 (4–15)	0.05
High IL-17A, >7.10 pg/mL *n (%)*	11 (15.7)	34 (30.1)	0.03

Quantitative variables are expressed in median and IQR (Interquartile range). Qualitative in frequency and percentage. SLEDAI: systemic lupus erythematosus disease activity, Anti-DNAds: anti-DNA double stranded. IL: interleukin. IFN-γ: interferon γ. Chi-square test was used for qualitative variables and Mann–Whitney U test for quantitative variables.

**Table 3:** Comparison of rs7574865 polymorphism genotypes in high and low interferon SLE patients.

Patients with SLE *n* = 206	High IFN-γ *n* = 73	Normal IFN-γ *n* = 133	*OR*	95% CI	*p*
Genotype rs7574865					
Genotype GG, *n* = 74 (%)	19 (26.0)	46 (34.6)	-	-	
Genotype GT, *n* = 110 (%)	34 (46.6)	66 (49.6)	-	-	0.11
Genotype TT, *n* = 48 (%)	20 (27.4)	21 (15.8)	-	-	
Recessive Model (TT vs. GG + GT)	-	-	2.01	1.01–4.03	0.046
Dominant Model (GT + TT vs GG)	-	-	0.67	0.35–1.25	0.21
Alleles, 2*n* = 412 G allele, 2*n* = 230	2*n* = 146 72 (49.3)	2*n* = 266 158 (59.4)	Reference		
T allele, 2*n* = 182	74 (50.7)	108 (40.6)	1.50	1.01–2.26	0.04

Qualitative variables are expressed in frequency and percentage. SLE: systemic lupus erythematosus. GG: wild homozygote genotype; GT: heterozygote genotype; TT: polymorphic homozygote genotype; OR: odds ratio. 95% CI: confidence interval 95%. IFN-γ: interferon γ.

**Table 4:** Factors associated with high IFN-γ in the logistic regression.

	Univariate into Method			Multivariate Stepwise Method		
Patients with SLE *n* = 206	*OR*	95% CI	*p*	*OR*	95% CI	*p*
Recessive Model (TT vs. GG + GT)	2.50	1.12–5.57	0.02	2.37	1.08–5.20	0.03
Disease duration, year median (IQR)	1.03	0.99–1.07	0.14	-	-	-
High IL-12, >0.52 pg/mL *n (%)*	2.78	0.87–8.89	0.08	-	-	-
High IL-17A, >7.10 pg/mL *n (%)*	2.21	1.008–4.84	0.04	2.52	1.16–5.47	0.02
Immunosuppressive drugs, *n (%)*	0.95	0.44–2.02	0.95	-	-	-

Multivariable logistic regression analysis. Dependent variable presence of high IFN-γ in SLE patients. OR: odds ratios; 95% CI: 95% confidence intervals. Crude ORs were obtained using the enter method. Adjusted ORs were obtained using the forward stepwise method.

## Data Availability

The datasets generated and/or analyzed during the current study are available from the corresponding author on reasonable request.
